# Discerning the spatio-temporal disease patterns of surgically induced OA mouse models

**DOI:** 10.1371/journal.pone.0213734

**Published:** 2019-04-11

**Authors:** Tobias Haase, Vikram Sunkara, Benjamin Kohl, Carola Meier, Patricia Bußmann, Jessica Becker, Michal Jagielski, Max von Kleist, Wolfgang Ertel

**Affiliations:** 1 Charité – Universitätsmedizin Berlin, corporate member of Freie Universität Berlin, Humboldt-Universität zu Berlin, and Berlin Institute of Health, Department of Traumatology and Reconstructive Surgery, Berlin, Germany; 2 Computational Medicine, Dep. of Numerical Mathematics, Konrad Zuse Institute, Berlin, Germany; 3 Systems Pharmacology and Disease Control, Dep. of Mathematics & Computer Science, Freie Universität Berlin, Berlin, Germany; Drexel University, UNITED STATES

## Abstract

Osteoarthritis (OA) is the most common cause of disability in ageing societies, with no effective therapies available to date. Two preclinical models are widely used to validate novel OA interventions (MCL-MM and DMM). Our aim is to discern disease dynamics in these models to provide a clear timeline in which various pathological changes occur. OA was surgically induced in mice by destabilisation of the medial meniscus. Analysis of OA progression revealed that the intensity and duration of chondrocyte loss and cartilage lesion formation were significantly different in MCL-MM vs DMM. Firstly, apoptosis was seen prior to week two and was narrowly restricted to the weight bearing area. Four weeks post injury the magnitude of apoptosis led to a 40–60% reduction of chondrocytes in the non-calcified zone. Secondly, the progression of cell loss preceded the structural changes of the cartilage spatio-temporally. Lastly, while proteoglycan loss was similar in both models, collagen type II degradation only occurred more prominently in MCL-MM. Dynamics of chondrocyte loss and lesion formation in preclinical models has important implications for validating new therapeutic strategies. Our work could be helpful in assessing the feasibility and expected response of the DMM- and the MCL-MM models to chondrocyte mediated therapies.

## Introduction

Osteoarthritis (OA) is one of the most common degenerative diseases of the musculoskeletal system affecting millions of people with a major loss in life quality. Due to the lack of regenerative capacity of the articular cartilage, OA is a progressive disease leading to increasing functional impairment of the joints. Despite intensive research in this field, the exact pathogenesis of OA is still not completely understood and remains an active area of investigation.

OA development in weight bearing joints has been associated with ageing, obesity, incorrect loading, and joint trauma. In addition to these, gender, genetic predisposition, and surgical interventions, are also known risk factors [1–4]. Furthermore, a state of chronic systemic inflammation that accompanies the metabolic syndrome is thought to contribute to OA development and progression [5]. In view of a rapidly ageing population combined with the epidemic of obesity and sedentary lifestyle, the prevalence of OA in the future is expected to rise [6].

Currently, treatments of OA are based only on palliative therapies that minimise pain symptoms; restrict movement; or at best slow down the course of the disease. To date, the progressive destruction of the articular cartilage and surrounding joint structures cannot be reversed or halted with these approaches [7]. Consequently, the disease inevitably progresses to a stage in which only surgical joint replacement provides a relief.

In OA, intrinsic and extrinsic factors form a complex regulatory network driving the progression into multifaceted phenotypes which include cartilage degradation, osteophyte formation, subchondral bone sclerosis, joint inflammation, and synovial fibrosis [8]. Therefore, an effective therapy in OA may need to target multiple factors simultaneously [9]. These challenges are currently being addressed with the design of disease modifying OA drugs (DMOADs) that directly alter disease factors [10]. For example, compounds that chemically block ECM degrading enzymes [11, 12] or anti-inflammatory drugs [13]. DMOADs were effective in altering their respective target factors and showed preclinical success, however, they did not translate into clinical practice yet [14–16]. It is widely accepted that this is likely due to the multifactorial nature of the disease. Overcoming this hurdle demands for therapies to target on a cellular level, the underlying principle being to target cells to regulate and steer the various OA factors in joints [17].

There are varying OA mice models each exploring different aspects of the disease; these models fall into the following categories: surgically induced, mechanically induced, spontaneous/genetically induced, and chemically induced. The prominent models used for assessing therapies are the surgically induced models. This is due to the fact that these surgical models mimic post traumatic OA (PTOA) in humans, for example cruciate ligament ruptures, meniscal tears, and subchondral bone sclerosis. The surgically induced models break down further into models specifically addressing either anterior cruciate ligament (ACL) transections or meniscectomies. Even though both types of injury are common among humans, it was shown that 50% of people who undergo a meniscectomy develop OA within 20 years from the day of the surgery [18]. Hence, meniscectomy based surgically induced OA models are being used in in vivo studies. In this work, we focus on two meniscectomy based methods: the Medial Collateral Ligament-Medial Meniscus transection model (MCL-MM) and the Destabilisation Medial Meniscus model (DMM). They are well established [19–28] and applied extensively in preclinical testing, however in order to reach translatability of chondrocyte targeting DMOAD therapies, deeper knowledge of chondrocyte population dynamics in these applied OA models is needed [29].

In light of this, we use computer vision to quantify chondrocyte- and apoptotic chondrocyte populations combined with standard histological evaluation to monitor multiple disease relevant parameters at various time points. Within each treatment arm, we assess the time point when these parameters change (so called change point analysis) to obtain insights into the spatial and temporal patterns underlying the OA models. Knowledge of the underlying dynamics is particularly useful to define the usability of either model for a specific therapeutic approach, especially with regard to choosing optimal time points for e.g. preclinical drug testing.

## Materials and methods

### Experimental animals and OA induction

All Animal studies were approved by the Landesamt für Gesundheit und Soziales Berlin (State Office of Health and Social Affairs Berlin, G0303/15) and were carried out in accordance with institutional and federal animal care guidelines. Male C57Bl/6J mice aged 11 weeks, weighing 20–24g were obtained from Charles River (Sulzfeld, Germany). Mice were housed under controlled conditions (22 ± 2 °C; 12h light-dark cycle) and allowed access to a regular sterile chow diet and water ad libitum. Two different models of surgically induced OA in the mouse knee joint were done according to the respective protocols described in Kamekura et al. (MCL-MM) [23] and Glasson et al. (DMM) [21]. Mice were anaesthetised with isoflurane (4% isoflurane at FiO2 = 1), and right hind limbs were shaved and prepared for aseptic surgery under a surgical microscope (Leica Microsystems). For the MCL-MM model, a skin incision at the medial side of the knee joint was done to visualise the medial collateral ligament. Using a blade, the medial collateral ligament was dissected and the joint capsule opened. Then the medial meniscus was visualised and carefully dissected without damaging the articular cartilage. Complete dissection of the meniscus was confirmed by dislocation of the meniscal parts in the joint. For the DMM model, a skin incision was done at the medial side of the patella. The patella was carefully dislocated to the lateral side and the capsule was opened. The meniscotibial ligament (MTL) was identified and dissected using a blade. Complete dissection of the MTL was confirmed by dislocation of the medial meniscus. After dissection of MM or MTL the joint capsule was flushed with saline, then the capsule and skin incision was closed with Vicryl sutures. Sham surgery was done to the right knees of sham mice similar to the MCL-MM surgery without dissection of the medial collateral ligament and meniscus. Since the MCL-MM sham procedure is likely to be more severe than the DMM sham procedure; we used it as a conservative candidate for baseline (control) measurements. Postoperative analgesia was maintained using carprofen (5mg/kg s.c. per day for 3 days post surgery).

### Experimental design, histological analysis and scoring

Mice of the MCL-MM, DMM and sham groups were sacrificed at 1, 2, 4, 6, 8 and 12 weeks post injury (in total 54 mice, n = 3 per group at each time point; see sample size selection outlined in S1 Methods. The complete right leg was dissected and fixed for 48 h at 4°C in paraformaldehyde to keep the knee joints in their natural position. The specimens were decalcified for 10 days in 14% EDTA at 4°C on a shaker. After dehydration, joints were embedded in paraffin and serial frontal sections (5μm) through the whole knee joint were done. Three sections (5μm) at 30μm intervals were stained with toluidine blue (TB) and used for scoring and morphometric analysis by a blinded observer. Cartilage destruction of the medial femoral condyle and tibial plateau was assessed using the established scoring systems of Glasson et al. [29] and Chambers et al. [30]. Histomorphometric measurements of the cartilage area, cartilage thickness (at minimal thickness), lesion width, and TB destained area were done as shown in the respective Figures using ImageJ software. Based on the measurements of periarticular excrescences, an osteophyte score was assigned as follows: 0 = up to 100 μm, 1 = 100 to 150 μm, 2 = 151 to 300 μm, and 3 ≥ 300 μm.

### Immunofluorescence staining

**Fig 1:**
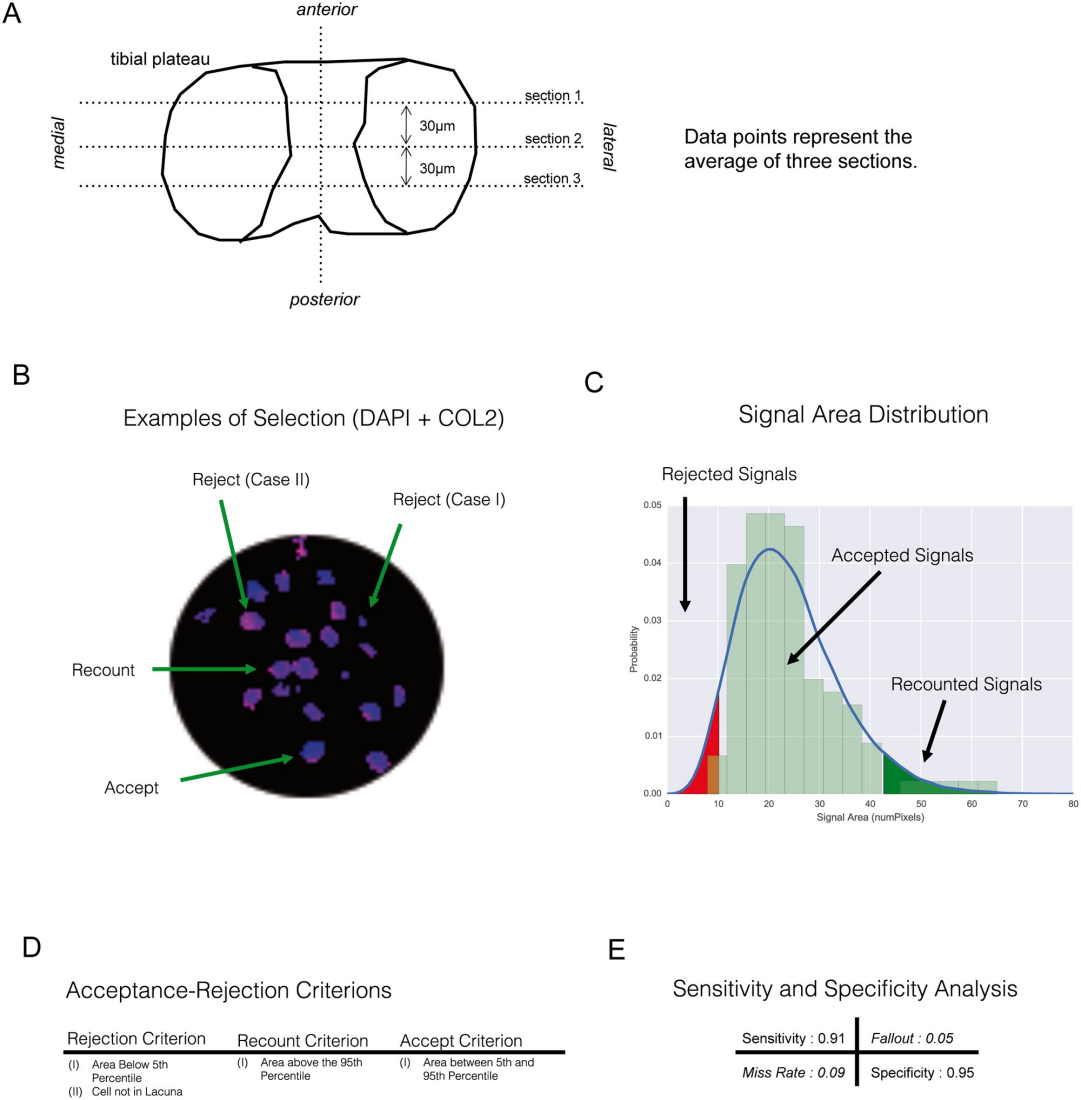
Computer assisted detection of chondrocytes and apoptotic chondrocytes in the histological sections of mouse knee joints. A) Example for the classification of different DAPI signals for the quantification of chondrocytes. B) Area distribution of accepted vs. rejected signals. C) Criteria that define acceptance and rejection of a signal. D) Sensitivity and specificity of the automated detection under the given criteria. E) Histological sectioning and analysis.

As described for the histomorphometric analysis, three frontal knee sections (5 μm) at 30 μm intervals (Fig 1A) were analysed by immunostaining with primary antibodies against collagen type I (COL1 A1/A2; Acris), type II (COL2 A2 [C-19]; Santa Cruz) and collagen cleavage products (Col2 3/4Cshort; IBEX Pharmaceuticals). The collagen type I staining was used for discrimination of articular cartilage and osteophytic cartilage structures (not shown). The collagen type II staining was used to discriminate the cartilages non-calcified from the calcified layer. Incubation with primary antibodies was followed by incubation with corresponding AlexaFluor-labelled secondary antibodies (Invitrogen) and DAPI staining of cell nuclei. Apoptosis in cartilage tissues was determined by TUNEL assay kit according to the manufacturer’s protocol (Roche Diagnostics).

### Image acquisition and processing

Fluorescent tissue sections were visualised using a confocal laser-scanning microscope (Leica TCS-SPE II). Images from the medial joint compartment were recorded as a merged z-stack of 10 scans through the tissue section plane using the same configurations in all scans. Computer vision was used for unbiased classification and quantification of positive DAPI and TUNEL signals in tissue images (Fig 1B–1E). The raw images went through four stages of processing, namely, zoning, thresholding, contouring, and classification. A detailed description of the steps is given in the S1 Methods.

### Statistical analysis

Our objective was to unravel critical dynamics in OA progression. For this, comparing samples between adjacent time points might not be statistically insightful. Generally, if the observed process is changing slowly, it is hard to distinguish adjacent sample populations with significance and consequently to identify critical time points where OA markers change. A more sophisticated approach for studying such behaviour would be to first partition the time course into phases using *change point analysis* and then—with the samples aggregated into the appropriate phases—investigate changes and differences between the respective phases. Herein, we used *change point analysis*, aggregated data into phases and compared markers within each investigated OA intervention as well as between different interventions. The performed change point analysis is described in S1 Methods.

## Results

### Histological assessment of OA development

**Fig 2:**
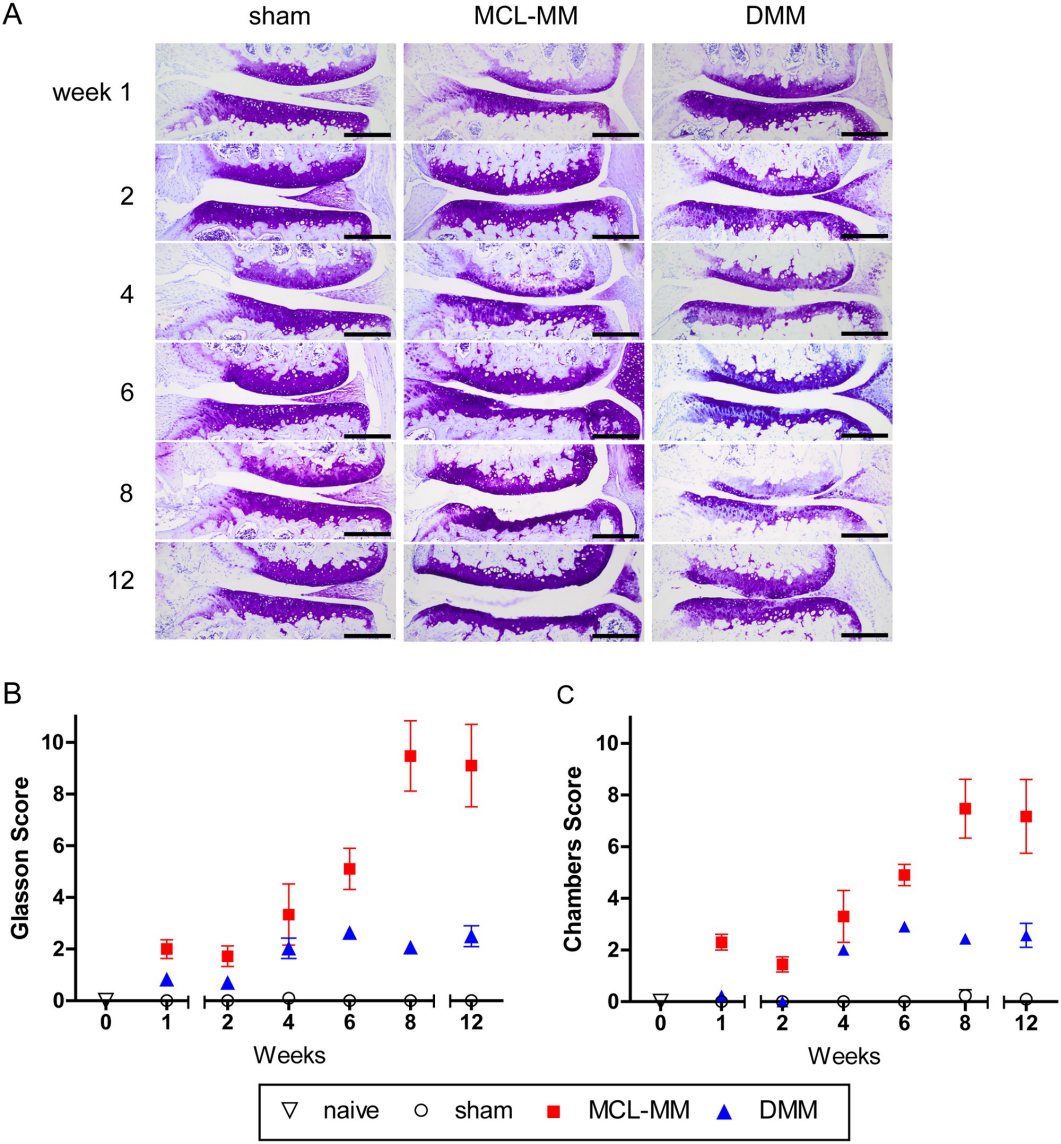
A Toluidine blue stained frontal sections of the medial aspect of mouse knee joints at the indicated time points after sham, MCL-MM, DMM surgery. OA-scoring of cartilage destruction of the medial joint aspect on the basis of the scoring systems of B) Glasson et al. and C) Chambers et al. Data represent mean values ± SEM. *p≤0.05 MCL-MM vs sham; ^$^p≤0.05 DMM vs sham; ^#^p≤0.05 DMM vs MCL-MM; Bar = 250μm.

Both surgical models (MCL-MM and DMM) resulted in a lateral extrusion of the medial meniscus compared to sham operated knee joints. This effect was observed during the surgical intervention and further verified in the histological sections of the knee joints (Fig 2A). As expected from the different modalities of the two surgical OA models, the histological examination showed that the extrusion of the medial meniscus was more pronounced in the MCL-MM model compared to the DMM model.

The accuracy of the surgeries was cross-validated to previous literature by assessing the degenerative changes in the mouse knee joints on serial frontal sections using the established scoring systems of Glasson et al. and Chambers et al. Both OA models induced progressive osteoarthritic changes compared to the sham operated mice (Fig 2). These degenerative changes were restricted to the medial aspect of the knee. The scores shown in all figures are the summarised values for the medial femoral condyle and tibial plateau. Evaluation of TB stained sections at sequential time points showed differences in OA progression and severity between the two models. In the MCL-MM model, the first changes in TB staining and clefts within the non-calcified cartilage zone became evident as early as one week post injury (Fig 2A). Although less pronounced, TB destaining was also visible in the central contact zone of the cartilage surfaces in the DMM model, an area that is in the following referred to as the weight bearing area. A further increase of the OA score became visible between four to six weeks post injury in both models. In the MCL-MM model, this increase was due to the beginning of lesion formation and progressive erosion of the non-calcified layer. At eight to twelve weeks after MCL-MM injury, major parts of the tibial plateau were eroded down to the calcified cartilage, in some cases down to the subchondral bone. In the DMM model, the TB destaining became more pronounced; occasionally roughened cartilage surfaces and vertical clefts were seen at four to six weeks post injury. However, there was no erosion of the cartilage until week 12 post injury.

**Fig 3:**
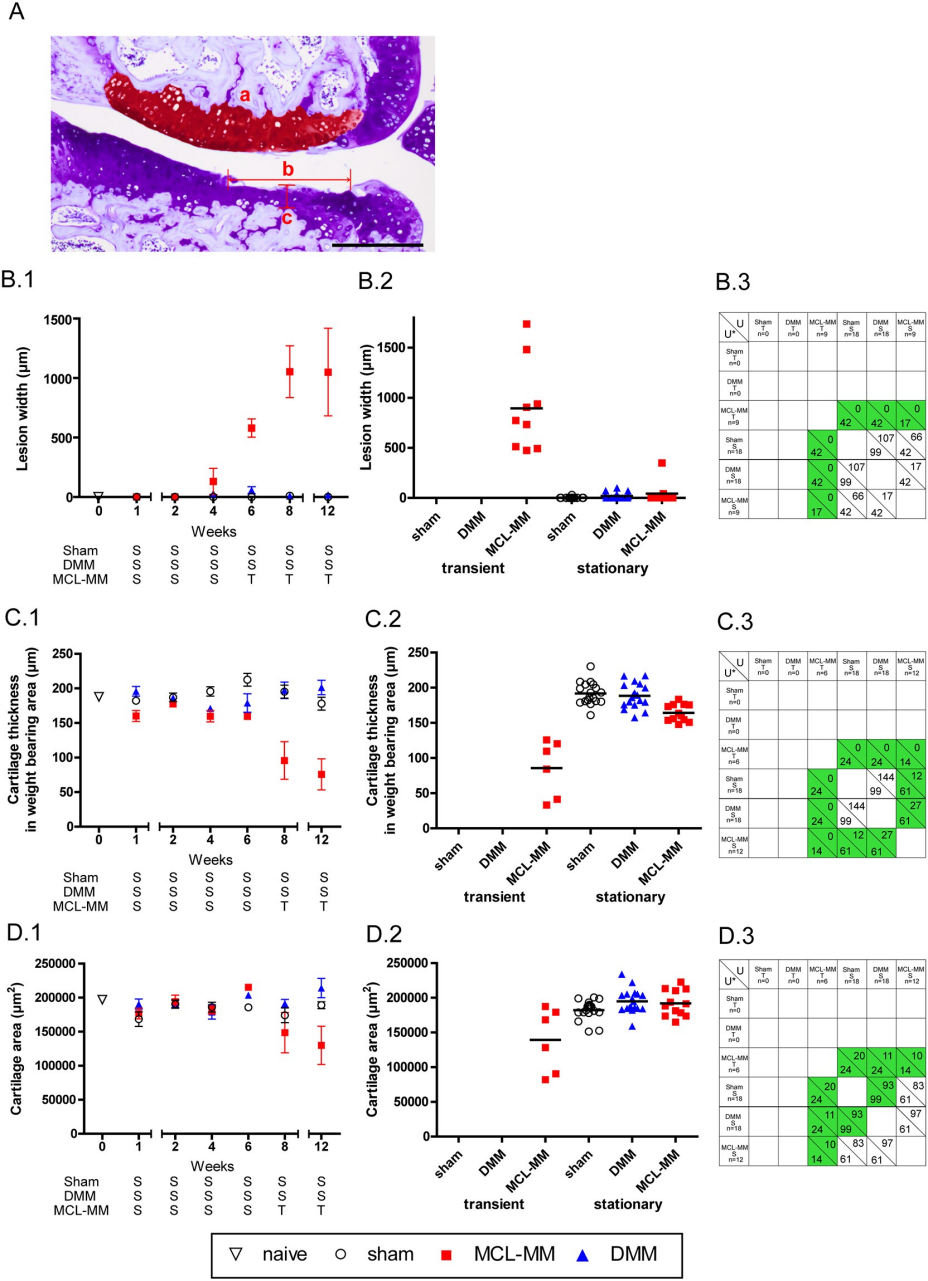
Assessment of progressing joint destruction on the basis of measurements on serial (A) TB stained joint sections of B) lesion width (b), C) cartilage thickness (c), D) cartilage area (a), B.1-D.1). The time course (mean ± SEM) of the studied effects (lesion width, cartilage thickness, cartilage area) in the surgical OA models of interest (sham, DMM, MCL-MM). The trend analysis for the classification of time points into stationary (S) and transient (T) phases is given in S1 Methods. Lesion width in surgical OA models aggregated into phases: sham (T) (none), DMM (T) (none), MCL-MM (T) (wks 6–12), sham (S) (wks 1–12), DMM (S) (wks 1–12), MCL-MM (S) (wks 1–4). C.2) Cartilage thickness in surgical OA models aggregated into phases: sham (T) (none), DMM (T) (none), MCL-MM (T) (wks 8,12), sham (S) (wks 1–12), DMM (S) (wks 1–12), MCL-MM (S) (wks 1–6). D.2) Cartilage area in surgical OA models aggregated into phases: sham (T) (none), DMM (T) (none), MCL-MM (T) (wks 8,12), sham (S) (wks 1–12), DMM (S) (wks 1–12), MCL-MM (S) (wks 1–6). B.3-D.3) Two-tailed Mann-Whitney U tests between the respective phases (transient, stationary) in the OA models (sham, DMM, MCL-MM). The number below the diagonal line in each cell shows the critical values U* for the corresponding sample sizes of the populations and α = 0.05. The number above the diagonal in each cell shows the test statistic U. The cells coloured green are showing statistical significance between the two populations (U ≤ U*). B.3 Lesion width analysis, C.3 Cartilage thickness analysis and D.3 Cartilage area analysis. Bar = 250μm.

Measurements of lesion width, cartilage thickness, and cartilage area were done to further assess and distinguish the processes of cartilage degeneration in both models (Fig 3A–3D). The serial TB stained joint sections revealed the formation of cartilage lesions starting after week four in the MCL-MM model (Fig 3B.1). The lesion width continuously increased affecting primarily the non-calcified layer until week eight. This led to minor changes in cartilage thickness in these cases. From week eight onwards, the cartilage erosion proceeded from the non-calcified layer down to the calcified cartilage, which led to an increased loss of cartilage thickness (Fig 3C.1). This pronounced cartilage degeneration is reflected by a loss of total cartilage area at week twelve in the MCL-MM model (Fig 3D.1). In contrast, there were no significant changes between DMM and sham with respect to lesion width and cartilage thickness (Fig 3B.2 and 3B.3, 3D.2 and 3D.3). Notably, some slices from the DMM operated mice, at later time points, did show a non-zero lesion score, however, this only occurred in one of the three slices analysed. There was no robust indication of lesion formation in the load bearing area in the DMM model (Fig 3B.1–3B.3). These results show that both models differ significantly in disease progression and disease severity.

### Chondrocyte populations and apoptosis in naive mice

**Fig 4:**
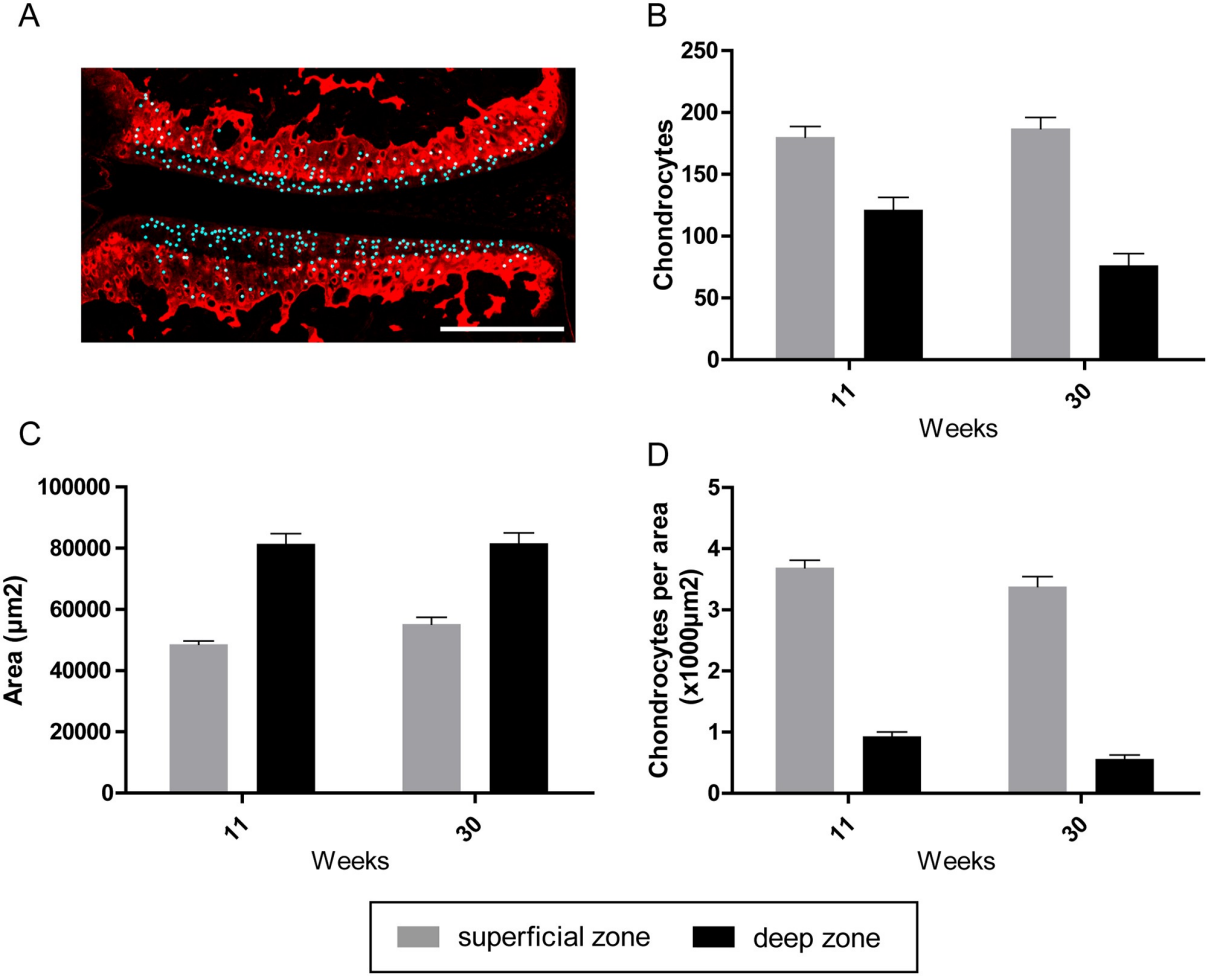
**Automated detection and quantification of chondrocytes in naive mice of 11 and 30 weeks of age (A)**. (B) Dynamics of chondrocyte numbers in the non-calcified zone and calcified zone. (C) Area of the non-calcified zone and calcified zone. (D) Chondrocyte numbers in the non-calcified zone and calcified zone. Data represent mean values ± SEM. Bar = 250μm.

Immunofluorescence staining with antibodies against collagen type II clearly separated the articular cartilage into the collagen type II rich calcified zone and the non-calcified zone with faint fluorescence signal (Fig 4A). Analysis of chondrocyte content within these two zones revealed that the cellular density in the non-calcified zone was four times higher than in the underlying calcified zone in naive mice (Fig 4B–4D). Chondrocytes of the non-calcified zone were evenly distributed from the medial to lateral aspect in frontal sections and displayed a flattened shape directly underneath the articular surface.

### Changes in chondrocyte population in OA development

**Fig 5:**
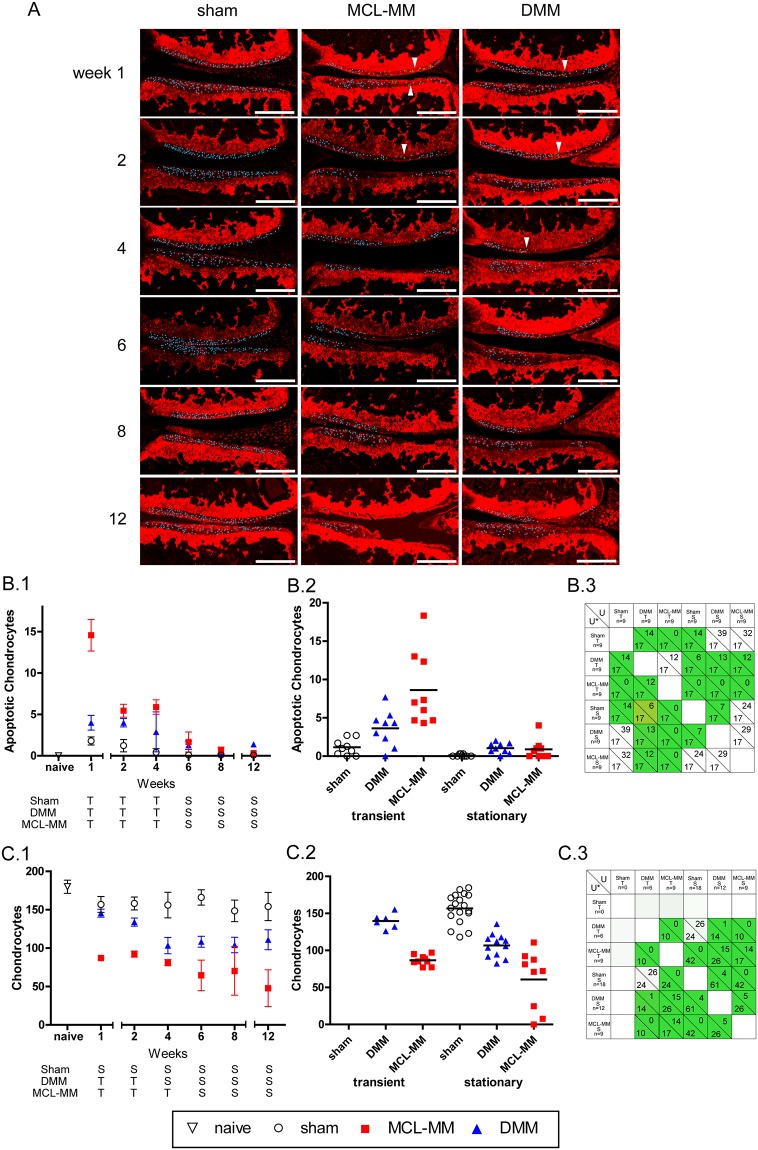
Automated quantification of chondrocyte numbers and apoptotic cells in the cartilage non-calcified zone of the tibial plateau and femoral condyle in different injury groups. A) Automatically detected and counted chondrocytes (blue) and apoptotic cells (green, arrow heads) in the medial aspect of the knee joint. Bar = 250μm. B.1-C.1) The time course (mean ± SEM) of the studied effects (average apoptotic chondrocyte population, average chondrocyte population) in the surgical OA models of interest (sham, DMM, MCL-MM). The trend analysis for the classification of time points into stationary (S) and transient (T) phases is given in S1 Methods. Apoptotic chondrocyte population samples in surgical OA models aggregated into phases: sham (T) (wks1-4), DMM (T) (wks1-4), MCL-MM (T) (wks 1–4), sham (S) (wks 6–12), DMM (S) (wks 6–12), MCL-MM (S) (wks 6–12). C.2) Chondrocyte population samples in surgical OA models aggregated into phases: sham (T) (None), DMM (T) (wks 1–2), MCL-MM (T) (1–4), sham (S) (wks 1–12), DMM (S) (wks 4–12), MCL-MM (S) (wks 6–12). B. 3-C.3) Two-tailed Mann-Whitney U tests between the respective phases (transient, stationary) in the OA models (sham, DMM, MCL- MM). The number below the diagonal line in each cell shows the critical values U* for the corresponding sample sizes of the populations and α = 0.05. The number above the diagonal in each cell shows the test statistic U. The cells coloured green are showing statistical significance between the two populations (U ≤ U*). B.3 apoptotic population analysis and C.3 chondrocyte population analysis.

In order to analyse the processes in OA initiation and progression, the viable- and the apoptotic chondrocyte population within the non-calcified layer of the femoral condyle and tibial plateau was quantified (Fig 5A–5C and S1 Fig).

Analysis of the TUNEL positive chondrocyte showed that all three groups (sham, DMM and MCL-MM) underwent both transient and stationary dynamics over the time course of investigation (12 weeks post injury). Specifically, all three groups went through an increase in the number of apoptotic chondrocytes, followed by a decay and then a plateau. The period of each group’s transient- and stationary phase were similar, however, the magnitudes of increase/decay were vastly different between the groups (Fig 5B.2). Spatially, chondrocyte apoptosis was narrowly restricted to the weight bearing area of the femoral condyle and the tibial plateau in both MCL-MM and DMM models (Fig 5A). Sham operated knees also showed occasional apoptotic chondrocytes, however, these cells were not isolated to the weight bearing area but were evenly distributed over the non-calcified zone. In the DMM model, chondrocyte apoptosis was less pronounced than in the MCL-MM model, but still more severe than in sham. In the transient phase, there were significant differences in the number of apoptotic chondrocytes between all groups. Notably, there was an approximate twofold increase in the mean number of apoptotic chondrocytes from sham to DMM to MCL-MM (Fig 5B.2). By week 6, the three models started showing a reduction in the number of apoptotic chondrocytes, with MCL-MM and sham not showing significant differences, due to the lack of chondrocytes in the non-calcified zone MCL-MM model. The DMM model on the other hand showed a significant difference compared to sham in the stationary phase (Fig 5B.2-3). This analysis implies that chondrocytes continued to undergo apoptosis the DMM model, whereas in the MCL-MM model, most chondrocytes had disappeared in the existing cartilage tissue.

The chondrocyte populations changed corresponding to the dynamics of apoptosis (Fig 5B.1 and 5C.1). The sham mice average chondrocyte population showed minor fluctuations in magnitude, but not significant enough to justify a time-trend (see *change point analysis* in S1 Methods Table B). In the DMM model, the average chondrocyte population was transiently decaying until week 4, followed by a plateau phase (Fig 5C.1). The MCL-MM model showed signs of having an early transient decay between week 0 and 1, followed by a plateau in the chondrocyte population post week 2. However, with the exception that after week 4, there was an approximate one in three chance of an MCL-MM operated mice to lose nearly all chondrocytes in their medial tibial plateau (Fig 5C.2). The loss in chondrocytes in this particular phase could be directly associated with the decrease in cartilage area and progression of lesion formation (Fig 3C.2 and 3B.2). The average chondrocyte population in the DMM and the MCL-MM operated mice were significantly different, with the MCL-MM mice having a lower population than the DMM operated mice. These results showed that there are significant differences in the chondrocyte population during early OA development between the two models (Fig 5C.2-3).

### Changes in ECM components in OA development

**Fig 6:**
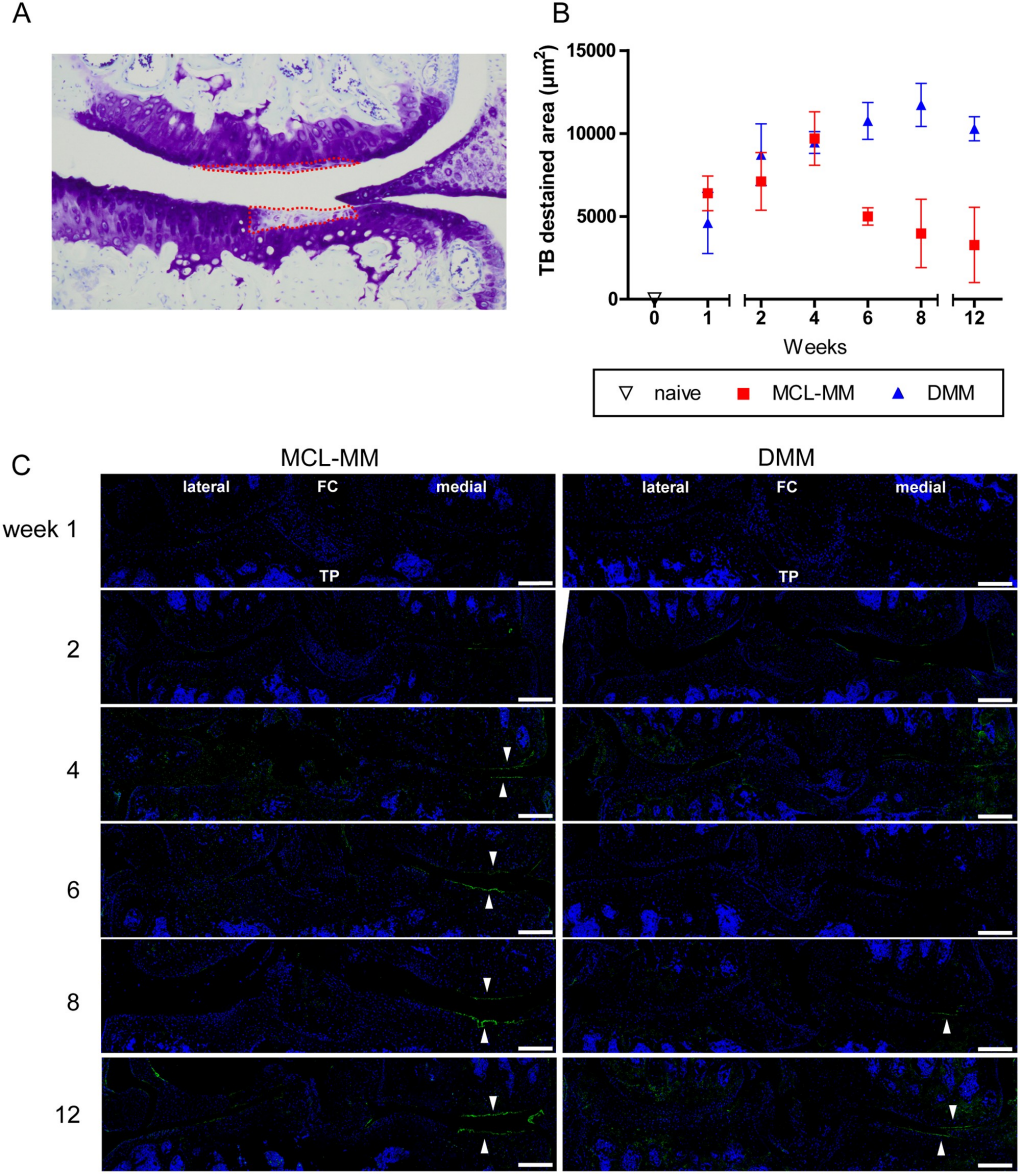
Alterations in cartilage proteoglycan- and collagen type II content in OA-progression. A) Quantification of toluidine blue destained areas on frontal sections showing the medial aspect of the knee joint. B) Dynamics of proteoglycan loss in the MCL-MM and DMM model. C) Immunofluorescent staining of collagen degradation products (green) and cell nuclei (blue) in MCL-MM, and DMM injured mice. Images show a frontal view on the lateral and medial aspect of the knee joint. Accumulation of collagen degradation products is detected at the cartilage surface in the weight bearing area (white arrow heads) in the medial aspect of the joint. Data represent mean values ± SEM. FC-femoral condyle, TP-tibial plateau, Bar = 250μm.

The first significant change that became visible after surgical destabilisation was a loss in TB staining, indicating proteoglycan (PG) destruction (Figs 2A and 6A). Closer examination of this effect revealed that early PG loss was restricted to the weight bearing area of the medial femoral condyle and the tibial plateau. Quantification of these destained areas showed that the rate of PG loss is similar in both models until week four (Fig 6A and 6B). Then, in the MCL-MM model a lesion is formed in the PG deficient area, whereas in the DMM model the PG loss is continuous until week twelve with no cartilage lesion development (S2A and S2B Fig).

Besides PGs, collagen type II is the main component of the articular cartilage extracellular matrix (ECM). Alterations in ECM composition, namely PG and collagen loss due to mechanical impact and catabolic enzymes, are assumed to be pivotal hallmarks of OA development and progression. Analysis of collagen type II abundance revealed no obvious changes in collagen type II content in the weight bearing areas, where the PG content was seen to have drastically decreased (Fig 5A).

Detection of collagen type II cleavage products revealed increased collagen degradation specifically on the cartilage surface in OA-induced joints (Fig 6C). Sham surgery did not induce increased collagen degradation at any time point (S3 Fig). Interestingly, collagen type II degradation was localised exclusively at the cartilage surface of the weight bearing area that was shown to be devoid of chondrocytes early after OA induction in the MCL-MM model (Fig 6C).

### Osteophyte development

**Fig 7:**
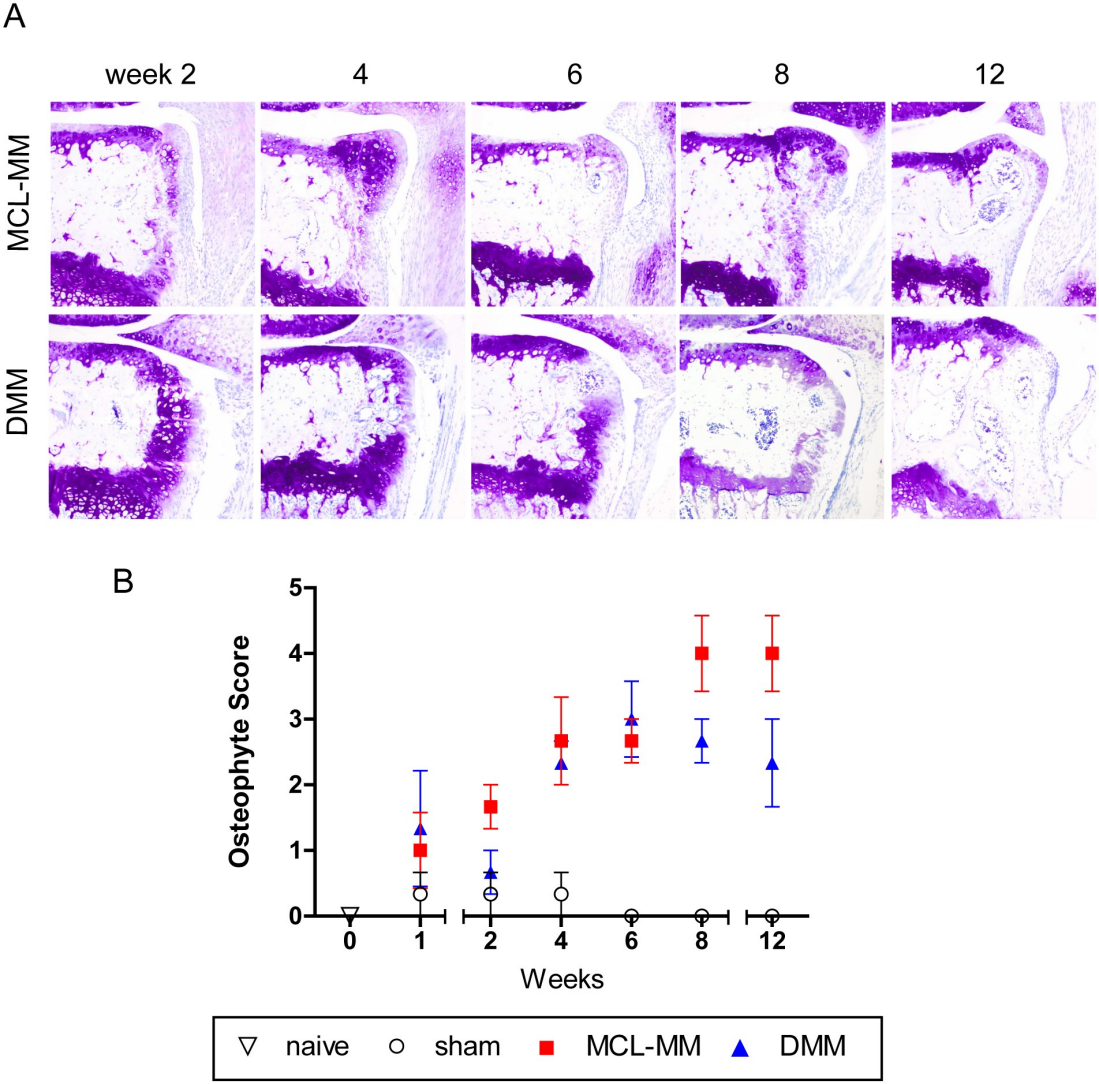
**Toluidine blue stained frontal sections of chondrophyte/osteophyte extensions at the medial tibial plateau after MCL-MM and DMM injury (A)**. (B) Assessment of osteophyte development at the medial femoral condyle and tibial plateau. Data represent mean values ± SEM. Bar = 250μm.

In addition to cartilage destruction, the second hallmark of osteoarthritis is the development of osteophytes in the periarticular region. Evaluation of osteophyte formation on the basis of TB stained frontal sections revealed the development of cartilaginous structures as early as one week post injury (Fig 7A and 7B). Similar cartilaginous growth visible in sham operated knees at one week post injury (not shown) indicated that this initial growth might be induced by the surgical intervention itself. However, during the following time points, chondrophyte/osteophyte formation increases in both OA models compared to sham operated knees. While chondrophyte/osteophyte dimensions increased similarly in both OA models, it is important to note that there were critical differences considering the location of the formed osteophytes between both models. While in the MCL-MM model osteophytes developed as lateral extension to the articular cartilage, osteophytes in the DMM model were formed below the articular cartilage at the level of the growth plate (Fig 7A).

## Discussion

**Fig 8:**
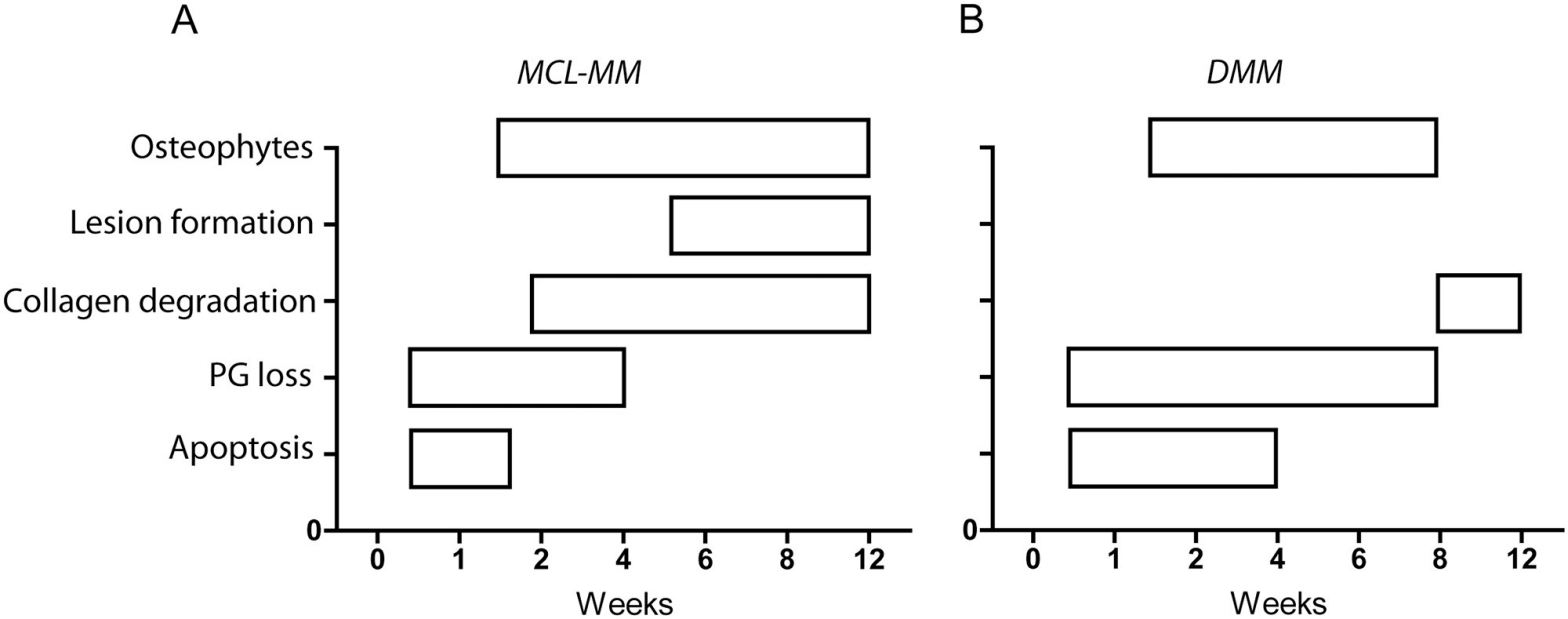
Proposed scheme that summaries the different processes in OA progression in a time resolved manner for the (A) MCL-MM and (B) DMM model.

In the present work we used a systematic approach combined with computer based automated analysis to discern the processes involved in OA progression within two widely used OA models. Analysis of various parameters at multiple time points after OA-induction allowed us to gain new insights into disease progression in these models (Fig 8). Our study was performed with a moderate number of animals (N = 54 animals in total, N = 3 animals per time point and surgical model, S1 Data). While this number may not suffice to naively compare adjacent time points, we chose a more suitable analytical method to analyse dynamics and time trends in the obtained data. Utilizing *change point analysis*, we could first identify critical timepoints where the dynamics of OA markers change in the different models. Subsequent pooling of data with identical dynamics (e.g. increase, decrease or stabilization of a marker) allows not only to conduct appropriate statistical comparison for the purpose of analysing time trends, but also allows to reduce the number of animals to be sacrificed.

Both models rely on the destabilisation of the fibrocartilaginous meniscus which provides load bearing and shock absorption functions in the knee joint. However, evaluation of serial TB stained histological sections at different time points post injury revealed that the two models differ vastly in quality and dynamics of the disease. TB staining of proteoglycans showed that in contrast to chemically induced OA models, osteoarthritic changes are narrowly restricted to the weight bearing area of the medial aspect of the knee joint. Although the surgically induced extrusion of the meniscus differs in both models, the area of impact is similar. This restriction to one confined area argues in favour of a solely mechanically induced initiation of OA in these models.

Proteoglycan loss is well known to be an early hallmark of OA progression [2]. However, the causal relationship between proteoglycan loss and chondrocyte apoptosis is not understood. From our experiments, we can conclude that proteoglycan loss and apoptosis occur in the same region, however, their progression occurs at different time points post injury. That is, MCL-MM and DMM show similar dynamics of proteoglycan loss, but different dynamics of chondrocyte apoptosis. Whereas proteoglycan loss and chondrocyte loss are paralleled in the DMM model, the loss of proteoglycans seems to occur on a slower timescale in the MCL-MM model until week six. The difference in PG loss dynamics between DMM and MCL-MM after week 6 was mostly due to the massive structural loss in MCL-MM. These observations may be explained by a *passive process*, in which chondrocyte depletion reduces the replenishment of proteoglycan, leading to its decay. However, a recent study [31], showed that proteoglycan loss does not occur in the absence of chondrocytes, pointing towards a more complex, not fully resolved mechanism, in which OA may trigger chondrocyte-dependent proteoglycan loss and chondrocyte apoptosis concurrently.

The staining for degradation products indicated collagen type II degradation to be particularly abundant in the proteoglycan deficient area. We observed that collagen type II degradation seems to be linked to lesion progression. On the basis of our results, therapies targeting collagen degradation should be administered two weeks, respectively eight weeks post injury in the MCL-MM vs. DMM model. Therapies targeting proteoglycan degradation have to be preventive or started at the time of OA induction in both models. Previous studies showed that there is a reversible ADAMTS mediated aggrecan degradation versus an irreversible MMP-mediated collagen degradation in OA [32–34]. The fact that there might be a reversible pathway suggests the possibility of cartilage healing, however, we have shown that the region where the proteoglycan loss occurs is devoid of cells. Hence, proteoglycan loss might be reversible, but cartilage healing is not probable given the lack of chondrocytes in that specific region.

Time resolved analysis of chondrocyte populations revealed that chondrocyte apoptosis in the non-calcified zone is an early event that is highly pronounced in the MCL-MM model. Again, apoptosis and following cell loss is restricted to the weight bearing area of the joint in both models, indicating a mechanically induced pathway that acts in a narrowly confined area. Importantly, cell numbers were constantly decreasing in the non-calcified zone leading to a continuously increasing cell free area. We were not able to detect an increase or accumulation of cells at the area of impact or its surroundings, indicating that migration or proliferation of stem- and progenitor cells did not take place in both models within the analysed timeframe.

Using an elegant approach, Zhang et al. showed that it is not the absence of chondrocytes but likely the catabolic phenotype of dying chondrocytes that induces cartilage degradation. They suggest that changes in the cartilage environment predisposes chondrocytes to adopt a catabolic phenotype that lead to cartilage degradation [27]. These observations are in line with the findings of Jeon et al., who could show that senescent cells in the joint are responsible for osteoarthritis progression [31]. It is tempting to speculate that early proteoglycan loss in the affected areas is a result of this chondrocyte catabolism. However, proteoglycan loss is evident in both models while the extent of cartilage destruction is different between them. In summary, there might be multiple (cellular, mechanical) factors that determine cartilage erosion.

Recently it was proposed that the location of osteophyte formation at the lateral joint regions differs depending on the underlying pathway. While BMP driven osteophytes develop in proximity of the growth plate, TGF-β driven osteophyte formation is located at the periosteum [35]. Interestingly, we observed that the osteophytes in the DMM model originate from the growth plate while the MCL-MM osteophytes located in direct proximity of the articular cartilage lateral to the joint space. In the DMM model osteophytes develop although there is nearly no cartilage loss, while in the MCL-MM model, due to cartilage destruction osteophytes become an active load bearing surface. This could indicate that the two models display different processes of osteophyte formation.

In our study, the MCL-MM model showed rapid OA progression with chondrocyte depletion and proteoglycan loss as early as one- to two weeks post injury. This is followed by rapid cartilage destruction and osteophyte formation. Early chondrocyte apoptosis is also a hallmark of non-invasive overload OA models [36] and its inhibition is explored as a potential therapeutic strategy in OA [37]. Using the MCL-MM model for validating chondrocyte targeting therapies is prone to fail if administered *after* OA induction given the lack of chondrocytes to act upon. The DMM model seems to be less severe, the chondrocyte numbers decrease more slowly and there is no lesion formation. However, the progressive cell loss in the weight bearing area in the DMM model will likely hamper cell targeting therapies if administered late after OA induction.

This study has elucidated that *time* is critical for chondrocyte targeting therapies in preclinical models. I.e., it is hard to judge the effectiveness of a chondrocyte targeted therapy when large areas of the cartilage are devoid of cells. Elaborating further, since the chondrocyte population is known over time, therapies whose effect is a function of the chondrocyte population can be qualified a priori. Given a time point at which the therapy is administered, the baseline efficacy can be determined and the necessary change to the baseline can be predicted. Lastly, we have shown the possibility of cartilage appear intact but the cartilage region being devoid of cells. Hence, when studying effectiveness of therapies in a pre-clinical setting, it would be prudent to also check the cartilage’s chondrocyte population.

A natural question and a limitation of our current study is whether the observed dynamics are also relevant for human OA, that is, does a patient’s cartilage also undergo a rapid cell loss and develop a cell free area and whether lesions also form in the cell free areas in human cartilage? Moreover, considering that we have seen that PG-degrading enzymes and collagen-degrading enzymes have different dynamics in the OA models, could the relative concentrations of these enzymes help explain the various disease progression seen in patients? In summary, chondrocyte targeting therapies seem to have a prospective outlook for modifying OA progression, however, therapies have to be precisely matched to the individual disease dynamics.

Our study has some limitations. First, we focused on two meniscus destabilization models that resemble the features of clinically relevant post-traumatic OA, however are likely based on similar mechanics. Overload- and enzyme-induced models probably display different phases and spatio-temporal dynamics. We did not analyse for changes in the microstructure or chemical composition of the extracellular matrix that may precede chondrocyte apoptosis and proteoglycan degradation [38]. Sophisticated studies by Das Neves Borges et al. [19] suggest that DMM injury induces elevated pressure forces, while the location of the contact pressure differs only slightly compared to control joints. We did not analyse for changes in loading force and loading location in the two models that might explain the different dynamics in OA progression.

## Supporting information

S1 Methods Change point analysis, sample size selection, zoning, thresholding and contouring, classification. (PDF)

S1 Fig Immunofluorescent staining of collagen type II (red), viable (DAPI, blue) and apoptotic chondrocytes (TUNEL, green) in frontal mouse knee sections at different time points. On the basis of these sections the computer assisted detection of chondrocytes and apoptotic chondrocytes was done (see Fig 5).
(TIF)

S2 Fig Comparison of proteoglycan loss and lesion formation in the MCL-MM (A) and DMM (B) model. (TIF)

S3 Fig Immunofluorescent staining of collagen degradation products (green) and cell nuclei (blue) in sham mice. Images show a frontal view on the lateral and medial aspect of the knee joint. FC-femoral condyle; TP-tibial plateau; Bar = 250μm.
(TIF)

S1 Data Underlying dataset. (XLSX)
