# Supplementary material for: Discerning the spatio-temporal disease patterns of surgically induced OA mouse models
Source: PLoS One. 2019 Apr 11;14(4):e0213734. doi: 10.1371/journal.pone.0213734 (PMC6459499; doi:10.1371/journal.pone.0213734)
Supplement: S1 Methods — (PDF) [file pone.0213734.s001.pdf]

Text A. Sample Size Selection

A type II/type I error ratio of 4 is typically recommended for experimental design, hence using $\alpha = 0.05$, a statistical power $(1-\beta)$ of greater than or equal to 80% is appropriate. We used the widely accepted Chambers score to determine the effect-and the sample size. Glasson et al. 2004 [1] report a baseline score $(mean_1 = 1.25,\ std_1 = 0.25)$ in Sham operated mice (Wk 4 & Wk 8) and a score for DMM $(mean_2 = 3.75, std_2 = 0.5$ at week $4)$, which we take as a minimal effect, see table below. Using these means and standard deviations, the effect size would be 6.06, which under a Wilcoxon-Mann Whitney one sided test requires a sample size of $N = 2$ for a power $(1-\beta)$ of 95%. These calculations are done a priori and form a guide for understanding the quality of our results a posteriori. For robustness, we choose $(N = 3)$.

Glasson et. al. 2004 (Chambers Score)

Week	Sham	Sham SEM	DMM	DMM SEM	Effect Size	Sample Size	Power
4	1	0.25	3.75	0.5	6.95	2	0.97
8	1.25	0.3	3.75	0.5	6.06	2	0.95

```
t tests - Means: Wilcoxon-Mann-Whitney test (two groups)

Options:    A.R.E. method

Analysis:   A priori: Compute required sample size
Input:      Tail(s)                        =  One
            Parent distribution            =  Normal
            Effect size d                  =  6.957011
            α err prob                     =  0.05
            Power (1-β err prob)           =  0.8
            Allocation ratio N2/N1         =  1
Output:     Noncentrality parameter δ      =  6.7984261
            Critical t                     =  3.1271576
            Df                             =  1.8197186
            Sample size group 1            =  2
            Sample size group 2            =  2
            Total sample size              =  4
            Actual power                   =  0.9796011
```

```
t tests - Means: Wilcoxon-Mann-Whitney test (two groups)

Options:    A.R.E. method

Analysis:   A priori: Compute required sample size
Input:      Tail(s)                        =  One
            Parent distribution            =  Normal
            Effect size d                  =  6.063391
            α err prob                     =  0.05
            Power (1-β err prob)           =  0.8
            Allocation ratio N2/N1         =  1
Output:     Noncentrality parameter δ      =  5.9251761
            Critical t                     =  3.1271576
            Df                             =  1.8197186
            Sample size group 1            =  2
            Sample size group 2            =  2
            Total sample size              =  4
            Actual power                   =  0.9502808
```

Table A: $G * Power$ output for the sample size calculation using the Glasson et al. 2004.

Text B. Zoning

The superficial zone was chosen as the area of interest as it was observed that the naive mice had near identical chondrocyte populations through time. Immunofluorescence stainings with specific antibodies directed against collagen type II were used to identify the region of articular cartilage. The clear and consistent sparsity of collagen type II staining in the upper cartilage layer was used to outline the superficial zone of the tibial plateau and femoral condyle. The superficial zone was considered the region of interest, and was used in the following for the automated analysis of chondrocyte and apoptosis populations.

Text C. Thresholding and Contouring

First, the image was thresholded on RGB such that any noise and light scattering due to the extracellular-matrix (ECM) in the image was removed. The DAPI and TUNEL signals were processed within their respective channels only. The thresholding of the respective channel was kept fixed for all images. Then any pixel which had an intensity above the threshold was amplified to the maximum intensity. Following this procedure, the

image should only contain regions where there is a possibility of a signal and regions of no signal. Then a contour was drawn around each connected set of pixels. Each contour was classified as a signal. The number of pixels within the contour was considered the area of the signal. The signal was then classified to be accepted, rejected or recounted (Fig 1).

Text D. Classification

The area of a true positive signal in the superficial zone in all images was assumed to follow the same contour area distribution. This distribution was empirically calculated by manually measuring the areas of 200 cells that are considered to be a true positive signal from randomly selected images. These sampled areas were then fitted to a gamma distribution. The accepted signal area begins within the 95 percentile confidence interval of the fitted gamma distribution. The extracted signals were then automatically assessed if they should be accepted, rejected or recounted (Fig 1). Within the processing of tissue sections it occurs that sometimes cell nuclei leave their natural position in the lacunae and are found in a region of collagen type II stained extracellular matrix. Since these nuclei cannot be clearly assigned to a specific lacunae or region, these signals were rejected and excluded from the analysis. These signals can be identified as they show an overlap of the DAPI or TUNEL staining and the collagen type II staining. In the case that separate nuclei are close to each other, the signal appears as the union of more than one nuclei. In such cases, if the signal area is greater than the 95th percentile, this signal is considered for recounting, by cutting them using the WaterShed method. All mentioned rules were applied to the images, so no human adjustments were needed to the data set generated by the automated process. To test the sensitivity and specificity of the data extraction method, ten slices were randomly selected from the pool of all images (excluding the images used for constructing the empirical distributions of the signal area) to compare the automated classification and human classification. The automated method had a sensitivity of 91% and a specificity of 95%.

Text E. Test Change Point Analysis

The aim is to split the time course into two phases: a *transient phase* and a *stationary phase*. The transient phase captures the studied effect changing, whereas, the stationary phase captures the studied effect stabilising. We distinguish these phases by detecting changes in the mean and variance through time. Specifically, in a transient phase we expect the means between adjacent time points to vary dramatically, whereas in the stationary phase, we expect the means among adjacent time points to be very similar. There may be more than two phases which the studied effect undergoes, however, in this work we consider modelling only two phases (we seek at most one change point). Finding a change point in a time series is referred to as a *binary segmentation*- or *At Most One Change* (AMOC) analysis in the *Change Point Analysis* literature [2]. We now formulate the problem statistically as done in [3]:

There are N time points which are ordered chronologically, and in each time point there are I replicates. Let μ_n, σ_n^2 be the mean and the variance of the studied effect at time n. Then the following hypotheses are considered:

$$
\begin{aligned}
H_0 \ &: \mu_1 = \mu_2 = \ldots = \mu_N = \mu \text{ and} \\
&\sigma_1^2 = \sigma_2^2 = \ldots = \sigma_N^2 = \sigma,
\end{aligned}
$$

versus the alternative hypothesis,

$$
\begin{aligned}
H_1 \ &: \mu_1 = \ldots = \mu_k \neq \nu_{k+1} = \ldots = \mu_N \text{ and} \\
&\sigma_1^2 = \ldots = \sigma_k^2 \neq \sigma_{k+1}^2 = \ldots = \sigma_N^2,
\end{aligned}
$$

where μ and σ^2 are unknown common parameters if there is no change, and k is the possible change point location. We use the *Schwarz Information Criterion* (SIC) as the test statistic for testing the hypotheses above. Before presenting the computational aspects of the hypothesis test, we describe the steps and principles of the analysis. The null hypothesis states that fitting a single Gaussian to all the samples is a better model than aggregating points on either side any k and fitting two Gaussians respectively. We denote $SIC(N)$ to be the Schwarz Information Criterion for fitting all samples to one Gaussian (H_0), and $SIC(k)$ the Schwarz Information Criterion of splitting the samples at time k and fitting two Gaussians respectively (H_1 for k). Hence, we have a series of alternative hypotheses; then to reject H_0 implies that there is no k, no splitting, which describes the samples better than a single Gaussian. More formally, the hypothesis test is given as follows:

$$\hat{k} := \underset{k \in \{2,\ldots,N-2\}}{\arg\min} \ SIC(k),$$

we fail to reject H_0 if

$$SIC(N) \leq SIC(\hat{k}) + c_\alpha,$$

and reject H_0 if

$$SIC(N) > SIC(\hat{k}) + c_\alpha.$$

The term c_α is the critical value from the distribution of $SIC(N)$, which was empirically calculated and given in [3, Table 1]. In our case $c_\alpha = 10.317$ for $\alpha = 0.05$ and the total number of samples is equal to 18. If H_0 is rejected, then the position $\hat{k}$ is a good estimate for the change point in the interval. We now present the details for calculating the test statistics described above. For $k \in \{2, \ldots, N-1\}$ we define:

$$\hat{\mu}_0 = \sum_{n=1}^{N}\sum_{i=1}^{I} \frac{x_n^i}{N\,I}, \qquad \hat{\mu}_{1,k} = \sum_{n=1}^{k}\sum_{i=1}^{I} \frac{x_n^i}{k\,I}, \qquad \hat{\mu}_{2,k+1} = \sum_{n=k+1}^{N}\sum_{i=1}^{I} \frac{x_n^i}{(N-k)\,I},$$

and

$$\hat{\sigma}_0^2 = \sum_{n=1}^{N}\sum_{i=1}^{I} \frac{\left(x_n^i - \hat{\mu}\right)^2}{N\,I}, \qquad \hat{\sigma}_{1,k}^2 = \sum_{n=1}^{k}\sum_{i=1}^{I} \frac{\left(x_n^i - \hat{\mu}_{1,k}\right)^2}{k\,I}, \qquad \hat{\sigma}_{2,k+1}^2 = \sum_{n=k+1}^{N}\sum_{i=1}^{I} \frac{\left(x_n^i - \hat{\mu}_{2,k+1}\right)^2}{(N-k)\,I},$$

where x_n^i denotes sample point of the ith replicate at time point n. The terms $\hat{\mu}_0$ and $\hat{\sigma}_0^2$ are the empirical mean and variance of all the samples; $\hat{\mu}_{1,k}$ and $\hat{\sigma}_{1,k}^2$ are the empirical mean and variance of the samples up to and including time k; lastly, $\hat{\mu}_{2,k+1}$ and $\hat{\sigma}_{2,k+1}^2$ are the empirical mean and variance of the samples from time points greater than k. Then the test statistics are calculated as follows: for H_0, the SIC for two unknowns is given by:

$$
\begin{aligned}
SIC(N) &= -2\log\left(L_0\left(\hat{\mu}_0, \hat{\sigma}_0\right)\right) + 2\log(N\,I), \\
&= N\,I\,\log(2\pi) + N\,I\,\log\left(\sum_{n=1}^{N}\sum_{i=1}^{I}(x_n^i - \hat{\mu}_0)^2\right) + N\,I + (2 - N\,I)\,\log(N\,I),
\end{aligned}
$$

where L_0 is the likelihood, that all samples are from a Gaussian distribution with mean $\hat{\mu}_0$ and variance $\hat{\sigma}_0^2$. Similarly, the SIC for the alternative hypotheses has four unknowns for each k and is given by:

$$SIC(k) = -2\log\left(L_1\left(\hat{\mu}_{1,k}, \hat{\mu}_{2,k+1}, \hat{\sigma}^2_{1,k}, \hat{\sigma}^2_{2,k+1}\right)\right) + 4\log(N),$$
$$= N\,I\,\log(2\pi) + (k\,I)\log(\hat{\sigma}^2_{1,k}) + (N-k)\,I\,\log(\hat{\sigma}^2_{1,k+1}) + N\,I + 4\log(N\,I),$$

where $L_1\left(\hat{\mu}_{1,k}, \hat{\mu}_{2,k+1}, \hat{\sigma}^2_{1,k}, \hat{\sigma}^2_{2,k+1}\right)$ is the likelihood, that samples up to and at time $t = k$ are from a Gaussian distribution with mean $\hat{\mu}_{1,k}$ and variance $\hat{\sigma}^2_{1,k}$, and samples from time greater than k are observed from a Gaussian distribution with mean $\hat{\mu}_{2,k+1}$ and variance $\hat{\sigma}^2_{2,k+1}$.

Table B: Change point analysis of the studied effects in the respective treatments. (*) The factor Lesion Width did not satisfy the required assumptions due to the nature of the variable, since a lesion is formed or not and a lesions only formed after a fixed time, trend analysis on Sham and DMM were omitted and we treat all the sample points as being from a single distribution.

		**Change Point**	**Critical Value**	**Test Statistic**	**Hypothesis Test**
**Studied Effects**	**Treatment**	$\hat{k}$	$SIC(\hat{k}) + c_\alpha$	$SIC(N)$	$SIC(N) > SIC(\hat{K}) + c_\alpha$
**Average Apoptotic Population**	Sham	4	41.933	52.110	**Reject H_0**
	DMM	4	79.031	83.071	**Reject H_0**
	MCLMM	4	103.602	114.466	**Reject H_0**
**Average Chondrocyte Population**	Sham	2	178.779	164.540	**Fail To Reject H_0**
	DMM	2	164.136	166.986	**Reject H_0**
	MCLMM	4	170.084	177.617	**Reject H_0**
**Average Lesion Width(*)**	Sham	N/A	N/A	N/A	**No Analysis**
	DMM	N/A	N/A	N/A	**No Analysis**
	MCLMM	4	266.069	282.281	**Reject H_0**
**Average Cartilage Thickness**	Sham	2	164.715	155.726	**Fail To Reject H_0**
	DMM	6	170.605	158.737	**Fail To Reject H_0**
	MCLMM	6	174.977	192.824	**Reject H_0**
**Average Cartilage Area**	Sham	8	413.080	399.569	**Fail To Reject H_0**
	DMM	8	415.596	406.457	**Fail To Reject H_0**
	MCLMM	6	435.788	436.178	**Reject H_0**

References

[1] Glasson SS, Askew R, Sheppard B, Carito BA, Blanchet T, Ma HL, et al. Characterization of and osteoarthritis susceptibility in ADAMTS-4-knockout mice. Arthritis and rheumatism. 2004;50(8):2547–58. doi:10.1002/art.20558.

[2] Aminikhanghahi S, Cook DJ. A survey of methods for time series change point detection. Knowledge and Information Systems. 2017;51(2):339–367. doi:10.1007/s10115-016-0987-z.

[3] Chen J, Gupta AK. Change point analysis of a Gaussian model. Statistical Papers. 1999;40(3):323–333. doi:10.1007/BF02929878.
