# Supplementary figures and images for: Discerning the spatio-temporal disease patterns of surgically induced OA mouse models
Source: PLoS One. 2019 Apr 11;14(4):e0213734. doi: 10.1371/journal.pone.0213734 (PMC6459499; doi:10.1371/journal.pone.0213734)

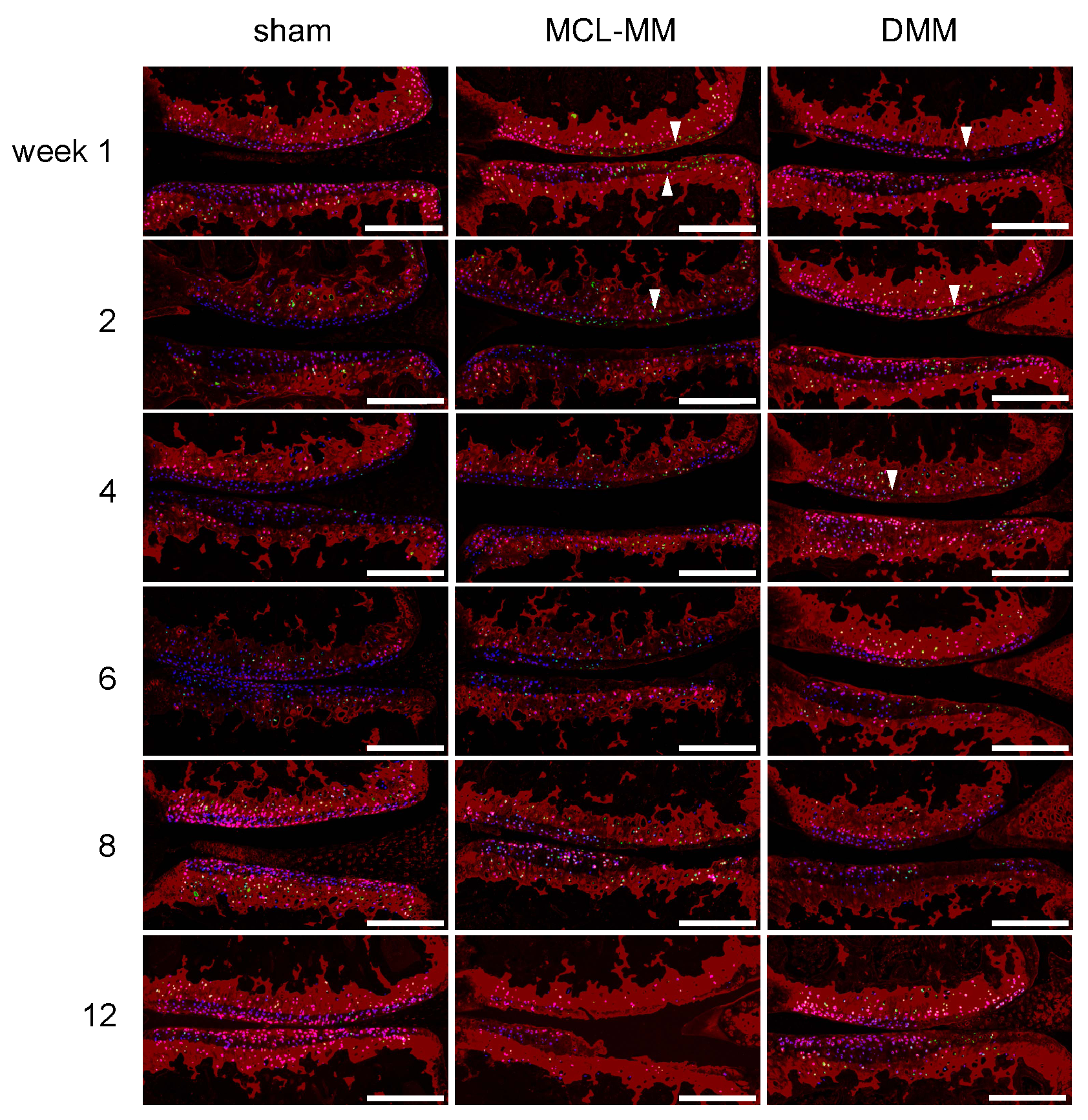

Supplement: S1 Fig — On the basis of these sections the computer assisted detection of chondrocytes and apoptotic chondrocytes was done (see Fig 5). (TIF) [file pone.0213734.s002.tif]

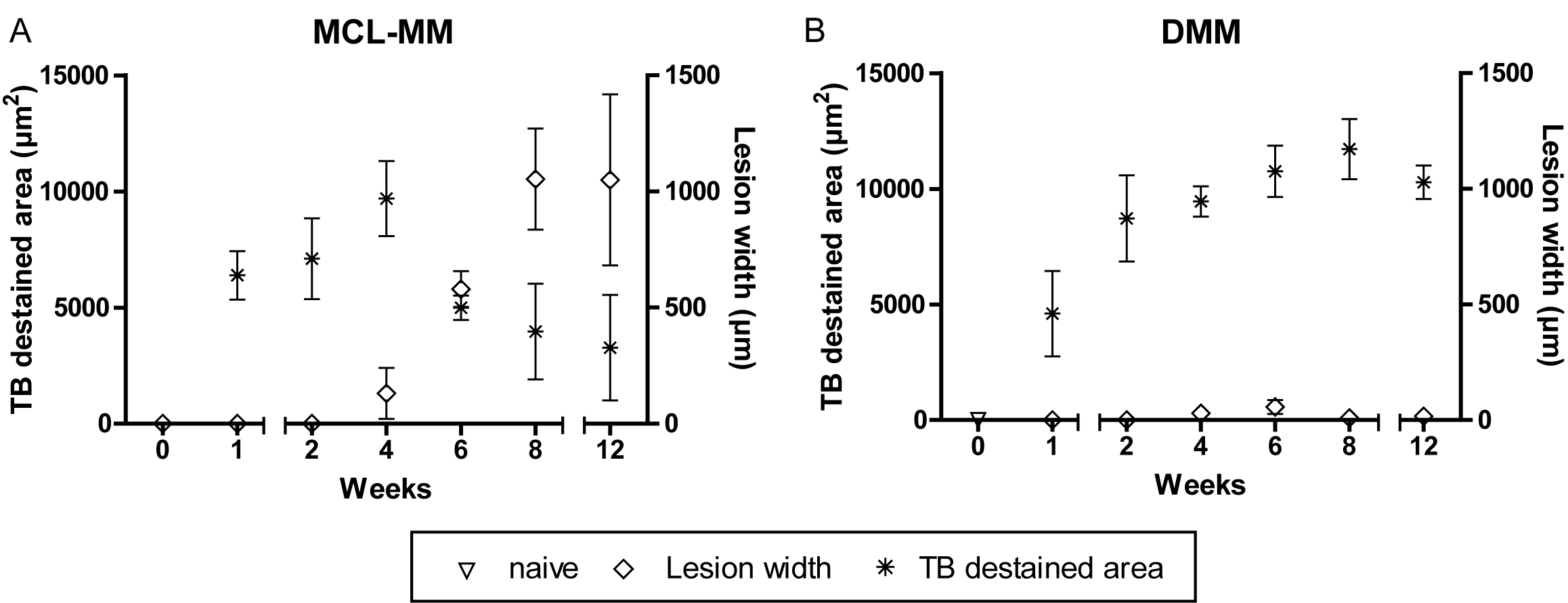

Supplement: S2 Fig — (TIF) [file pone.0213734.s003.tif]

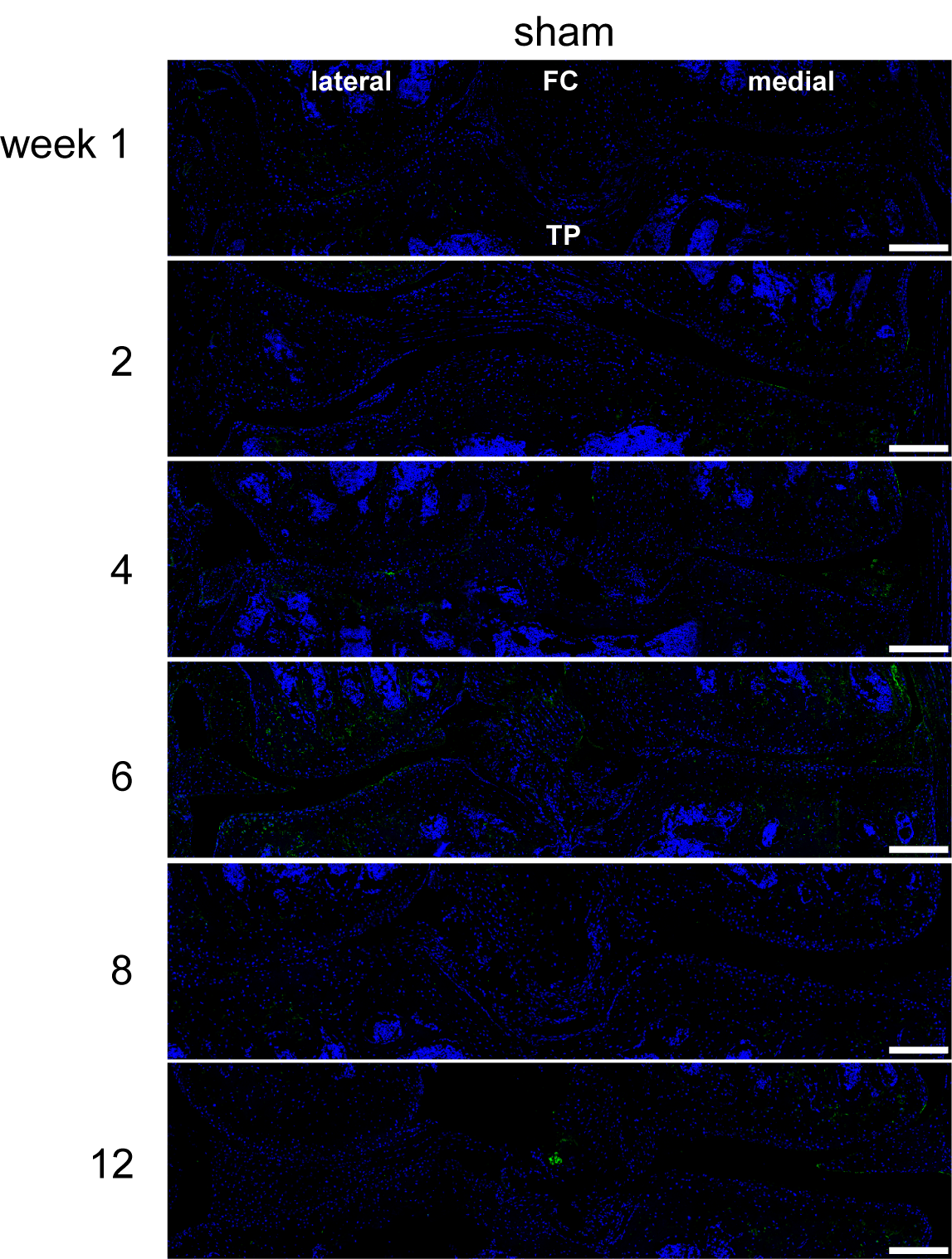

Supplement: S3 Fig — Images show a frontal view on the lateral and medial aspect of the knee joint. FC-femoral condyle; TP-tibial plateau; Bar = 250μm. (TIF) [file pone.0213734.s004.tif]
